# Potential protective efficacy of biogenic silver nanoparticles synthesised from earthworm extract in a septic mice model

**DOI:** 10.1186/s12896-024-00901-1

**Published:** 2024-10-11

**Authors:** Sara Bayoumi Ali, Ayman Saber Mohamed, Marwa Ahmed Abdelfattah, Alia Baher Samir, Farha Youssef Abdullah, Halla Ahmed Elsayed, Manar Abdelhalem, Nour Elsadek, Sara Osama, Seif Eldin Mohamed, Sohair R. Fahmy

**Affiliations:** Zoology Department, Faculty of Science, Cairo University, Giza, Egypt

**Keywords:** Ag NPs, Sepsis, Earthworm extract, Nanoparticles, Mice, Faeces

## Abstract

Sepsis is an inevitable stage of bacterial invasion characterized by an uncontrolled inflammatory response resulting in a syndrome of multiorgan dysfunction. Most conventional antibiotics used to treat sepsis are efficacious, but they have undesirable side effects. The green synthesised Ag NPs were synthesized by 5 g of the earthworm extract dissolved in a volume of 500mL of distilled water and then added to 2,500 mL aqueous solution of 1mM silver nitrate at 40 °C. After 4 h, the mixture was then allowed to dry overnight at 60 °C. Later, Ag NPs were washed and collected. They were characterized by X-ray diffraction, ultraviolet-visible spectroscopy, and transmission electron microscopy. Sepsis model as induced by feces-intraperitoneal injection method. Eighteen male mice were assigned into three main groups: the control group, the sepsis-model group, and the Ag NPs-treated group. The control group received a single oral dose of distilled water and, after two days, intraperitoneally injected with 30% glycerol in phosphate buffer saline. The Sepsis-model group received a single oral dose of distilled water. Ag NPs - The treated group received a single oral dose of 5.5 mg/kg of Ag NPs. After two days, the sepsis-model group and Ag NPs-treated group were intraperitoneally injected with 200 µL of faecal slurry. Ag NPs treatment in septic mice significantly decreased liver enzyme activities, total protein, and serum albumin. Moreover, Ag NPs significantly enhanced kidney function, as indicated by a significant decrease in the levels of creatinine, urea, and uric acid. In addition, Ag NPs showed a powerful antioxidant effect via the considerable reduction of malondialdehyde and nitric oxide levels and the increase in antioxidant content. The histopathological investigation showed clear improvement in hepatic and kidney architecture. Our findings demonstrate the protective efficacy of biogenic Ag NPs against sepsis-induced liver and kidney damage.

## Introduction

Sepsis is unequivocally defined as a severe systemic dysfunction resulting from the body’s uncontrolled response to infection [1]. According to the World Health Organization (WHO), 49 million cases and 11 million sepsis-related deaths were reported in 2017, making sepsis responsible for almost 20% of global deaths annually [2]. Sepsis is firstly characterised by the presence of a microbial infection that is accompanied by increased body temperature and heart rate. If left untreated, sepsis can progress to severe sepsis, where it is associated with several organ dysfunction as well as sepsis-induced hypotension. The final and most alarming stage of sepsis is known as “septic shock.” In this stage, patients experience persistent hypotension despite sufficient fluid resuscitation because of major perfusion abnormalities in their blood vessels [1]^,^ [3].

Current treatments for sepsis include antibiotics, which are decided after appropriate cultures have been obtained, vasopressors, and fluid therapy [4]. Given that antibiotics are the primary and only medication provided for patients with sepsis, this has majorly contributed to the growing concern of antimicrobial resistance (AMR), which is now regarded as an independent risk factor of its own [5]. The pathophysiology and treatment of sepsis have been studied using a variety of animal models with different induction techniques. The cecal ligation and puncture (CLP) model is currently regarded as a gold standard for sepsis studies. However, several limitations arise when adopting this approach, such as the high level of researcher variation and the challenge of comparing animals with various cecum shapes and sizes [6]. A study by Fang et al. in 2020 compared the CLP method against a new approach called faeces-intraperitoneal injection (FIP). This study concluded that the FIP model was way easier to establish, overcomes the variability in the CLP method, and offers a more standardised approach [7]. The increasing rate of bacterial resistance to antibiotics has propelled the search for alternative antibacterial agents. The recent advancements in nanotechnology have revealed the antimicrobial properties of various metal nanoparticles (NPs) [8]. Contrary to antibiotics, NPs are less prone to bacterial resistance due to the array of mechanisms by which they simultaneously exhibit their antibacterial activity. Among those mechanisms is the release of positive ions from NPs, targeting negatively charged lipopolysaccharides in bacterial cell membranes. This high-affinity interaction increases the rate of entry of ions into the bacterial cell, interrupting its process and internally damaging it. Ag + ions released from silver nanoparticles (Ag NPs) are especially destructive to proteins and enzymes rich in cysteine. Ag NPs bind to them irreversibly, breaking disulfide bonds and thus disrupting protein integrity and function [9]. Additionally, Ag NPs can accumulate on the bacterial cell surface and cross through the cytoplasm, leading to the denaturation and perforation of the cell membrane and damaging bacterial organelles [10].

Hence, Ag NPs were chosen as the anti-bacterial agent in this study, testing their efficacy against bacterial infections that induce sepsis. There are different physical and chemical methods for Ag NPs synthesis [11]. However, biogenically synthesized NPs are more easily prepared, cost-effective, less environmentally toxic, and more biocompatible and stable [12]. Earthworm extract has bioactive compounds that act as antibacterial and anticoagulant agents, such as Lumbricin-1 and lumbrokinase enzyme, in addition to their anti-inflammatory and antipyretic activities [13]^,^ [14]. The variety of functional groups in the extract is attributed to its chemically reducing property, making it a biocompatible reducing agent suitable for the biosynthesis of Ag NPs [15]. The *Allolobophora caliginosa* earthworm species, belonging to phylum Annelida, is the most abundant and commercially available in Egypt; it was even used as an edible source of protein in former times [16]. When applied in clinical settings, the biosynthesised silver nanoparticles (Ag NPs) have the potential to serve as a pretreatment for patients who may develop sepsis in the future. Consequently, the present study seeks to synthesize Ag NPs by using *A. caliginosa* earthworm extract and administering it to mice with sepsis induced by injection of faecal slurry.

## Materials and methods

### Collection and extraction of *Allolobophora Caliginosa*

*Allolobophora caliginosa* earthworms (1 kg) were purchased from commercial vermiculture in Cairo governorate and placed in paper bags with the sustenance of waterlogged soil. *Allolobophora caliginosa* earthworms were cleaned with tap water to remove debris and then were soaked for six hr. in distilled water. *Allolobophora caliginosa* were dissected into small particulates and placed in a glass beaker to deposit in an (80%) ethanol solution for two days in the refrigerator. This mixture was filtered and centrifuged for 10 min at 3000 rpm till it was clear. The supernatant was left to evaporate at (40℃) in a water bath for five hr., then further dried in the oven [17].

### Synthesis of biogenic silver nanoparticles

5 g of the *Allolobophora caliginosa* extract was dissolved in a volume of 500 mL of distilled water. The mixture was then added dropwise to 2,500 mL aqueous solution of 1mM silver nitrate (AgNO_3_) on the magnetic stirrer at 40 °C. After 4 h, the silver nitrate solution turned from transparent to brown, indicating the formation of Ag NPs. The mixture was then allowed to dry overnight in an oven at 60 °C. Later, Ag NPs were scratched, collected in falcon tubes, and washed with distilled water and methanol [18].

### Characterization of biogenic ag NPs

#### Ultraviolet-visible spectroscopy

The optical property of green synthesised Ag NPs was observed from the absorption spectra of nanoparticles synthesised at various temperatures and concentrations. UV–Vis spectra were measured using Varian; Cary 5000 UV-visible spectrophotometer with a wavelength in the range of 200–800 nm at room temperature [19].

#### X-ray diffraction analysis

Crystallographic properties of Ag NPs were explored using a PANalyticalX’Pert X-ray diffractometer equipped with a nickel filter using copper (Cu) Ka radiation as an X-ray source. The size of the particles was calculated using Scherrer’s formula as follows: d = Kλ/β cosθ. Where d is the crystalline size, K = 0.89 is the shape factor, k is the X-ray wavelength of Cu Ka radiation (0.154 nm), θ is the Bragg diffraction angle, and β is the full width at half maximum of the respective diffraction peak [20].

#### Transmission electron microscopy (TEM)

The morphological study of the green synthesised Ag NPs was performed by TEM, which was achieved at an accelerated voltage of 120 kV (JEM- JEM 2100 F; JEOL Ltd, Tokyo, Japan) (Electron Microscopy Unit, Faculty of Agriculture, Cairo University) [21].

### Acute toxicity test LD 50

The Median lethal dose (LD_50_) of Ag NPs was determined in accordance with the method described by (Chinedu et al.,2013) [22]. Eight Swiss albino mice were divided into four different groups (*n* = 2). Each group was gavaged with a different dose of Ag NPs. The doses were 10, 100, 300, and 600 mg/kg. After the administration, animals were observed for 1 h and again for 10 min every 2 h interval for 24 h. Signs of systemic toxicity were observed, including changes in (skin, ears, fur, eyes), piloerection, weakness, weight loss, food and water consumption, breathing difficulties, and death. From the following formula, LD_50_ was calculated: LD_50_ = (M0 + M1)/2. M0 is the highest dose of Ag NPs, which did not cause mortality, and M1 is the lowest dose of Ag NPs, which resulted in mortality [22].

### Determination of antibacterial minimal inhibitory concentration (MIC) of ag NPs

The inhibitory effect of Ag NPs on the growth of the test organisms was studied using MIC assay. The MIC of Ag NPs was identified to determine the lowest concentration that inhibits the visible growth of the test organisms [23]. *Staphylococcus aureus*, *Listeria monocytogenes*, *Pseudomonas aeruginosa*, and *Salmonella typhimurium* were used to test antibacterial activity. Briefly, bacterial suspensions were prepared as previously described under disc diffusion test, then diluted 1:1000 to give (1–2 × 105 CFU ml − 1). Tested bacteria were grown on nutrient broth in a shaking incubator (150 rpm). MIC values of tested nanoparticles were determined using the twofold serial dilution technique at concentrations ranging from 0.00267 to 0.428 mg ml − 1 for Ag NPs. The cultures were incubated at 37 °C for 18–20 h. Results were estimated by measuring optical density (OD) at 600 nm.

### Experimental animals

Eighteen CD1 (Mus musculus) adult male mice were obtained from the National Research Center (NRC, Dokki, Giza). Their weights ranged from 25 to 30 g. Prior to the experiment, mice were allowed to acclimate for seven days. Mice general conditions were observed once daily throughout the acclimatization period. Food and water were available ad libitum.

### Animal housing

Mice were grouped and housed in conventional polypropylene cages (6 per cage) and placed in the well-ventilated animal house of the Zoology Department, Faculty of Science, Cairo University, with a proper temperature of 23–26 °C, humidity of 50–60% and a 12 h/12 h light-dark cycle.

### Determining the sepsis induction dose by faeces-intraperitoneal injection (FIP)

Sepsis induction was done by faeces intraperitoneal injection. Faecal slurry stock preparation was performed as described by [24]. Ten grams of faeces were collected and sequentially added to 50 ml distilled water. The slurry was filtered and then soaked in an equal volume of 30% glycerol dissolved in phosphate buffer solution (PBS). For the purpose of determining the sepsis induction dose, eight male mice were divided into four groups (2 mice/group). Group 1, which was the control, was injected with a PBS-glycerol solution. A series of different faecal slurry doses of 100, 200, and 300 µL was prepared and administered intraperitoneally to three distinct groups of mice. The last dose of 100 µL resulted in only 50% mortality after six days from the injection day. By increasing the dose to 200 µL, the mortality increased to 100% after seven days. A dose of 300 µL resulted in 100% mortality; however, the death was after two days only. In accordance with the experiment duration, the dose of 200 µL was determined to induce sepsis in mice and can be used in the experimental design.

### Experimental design

**Fig. 1:**
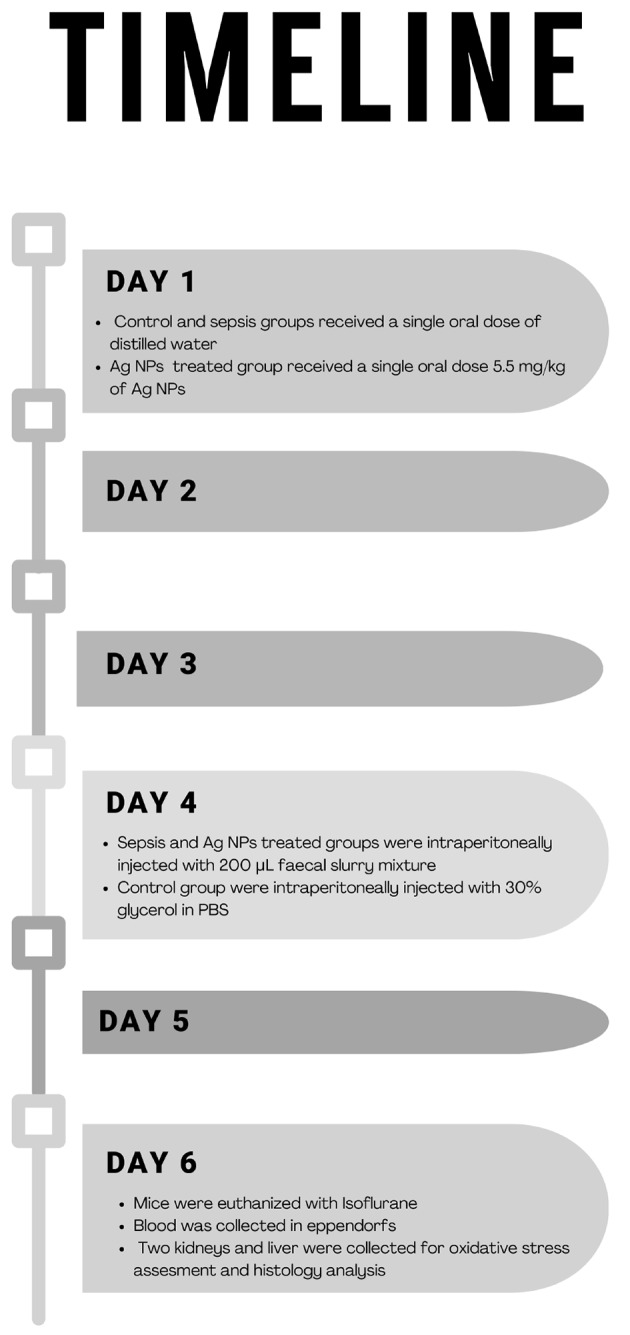
Timeline of the experimental period

Eighteen male mice were divided into three groups (6 mice in each group), and the treatment started two days before faecal intraperitoneal injection as follows: Group 1 (control group): mice received a single oral dose of distilled water, and after two days, they were intraperitoneally injected with 30% glycerol in PBS. Group 2 (sepsis model group): mice received a single oral dose of distilled water, and after two days, they were intraperitoneally injected with a 200 µL faecal slurry mixture. Group 3: (Ag NPs - treated group): mice received a single oral dose of 5.5 mg/kg of Ag NPs, and after two days, they were intraperitoneally injected with a 200 µL faecal slurry mixture (Fig. 1).

### Sample collection

After six days, mice were euthanized with Isoflurane. Afterward, blood was collected in Eppendorf and centrifuged for 10 min at 3000 rpm for the separation of sera, which was then stored at -80 °C for the biochemical parameters assessment. The two kidneys and liver were dissected from each mouse and immediately placed in saline, then on filter papers so as to remove any blood traces. For each mouse, a kidney and half a liver were stored at -80 °C for the determination of oxidation stress markers. The remaining parts of the liver and kidney were fixed in 10% formal saline to examine histopathology in both organs.

### Biochemical parameters assessment

Levels of alanine aminotransferase (ALT), aspartate aminotransferase (AST), alkaline phosphatase (ALP), albumin, creatinine, urea, and uric acid in the serum were measured according to the instructions of the manufacturer, using Bio-Diagnostic kits (Giza Governorate, Egypt).

### Oxidative stress markers

The kidney and liver homogenate were centrifuged, and the supernatant was used for the biochemical analysis and determination of reduced glutathione (GSH), glutathione-s-transferase (GST), malondialdehyde (MDA), catalase (CAT), nitric oxide (NO), and superoxide dismutase (SOD) according to the instructions of the manufacturer, using Bio-Diagnostic kits (Giza Governorate, Egypt).

### Histopathological examination

After the liver and kidney tissue specimens were collected, they were promptly fixed in 10% neutral buffered formalin, and then they were processed using the conventional histological procedures. Through the utilization of a microtome, tissue blocks that were fixed in paraffin were sectioned in order to acquire sections that varied in thickness from 5 μm to 10 μm to 15 μm. It was made sure that the techniques used to section all the specimens were consistent with one another in order to reduce the amount of variation.

The H&E staining was carried out using previously established techniques, with some minor adjustments.

In a nutshell, the sections that had been embedded in paraffin were deparaffinized in xylene and then rehydrated by means of a successively increasing series of alcohol solutions. After that, the slices were submerged in Harris Hematoxylin solution to stain the nuclei. This was then followed by differentiation in acid alcohol and bluing in Scott’s tap water. Following the rinsing process, the sections were counterstained with eosin solution in order to visualize the components of the cytoplasm. In order to conduct a microscopic examination, stained sections were first dehydrated, then cleaned, and then mounted with a [25].

### Statistical analysis

All data were expressed as mean ± standard deviation (SD). The comparisons within groups were tested using a one-way analysis of variance (ANOVA) with the Duncan post hoc test, and *P* < 0.05 was considered statistically significant. SPSS for Windows (version 15.0) was used for the statistical analysis.

## Results

### Characterization of biogenic ag NPs

#### Transmission electron microscope

**Fig. 2:**
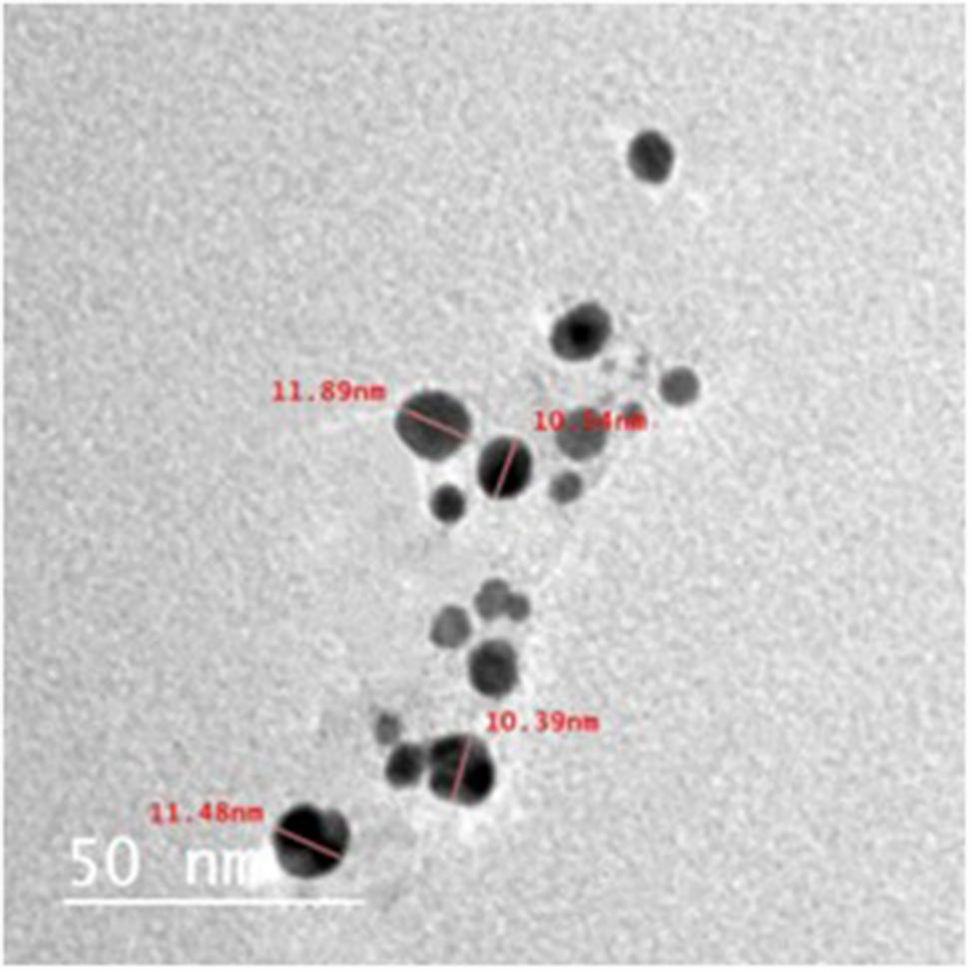
TEM micrographs showing the structure and size of biosynthesized Ag NPs using earthworm extract

**Fig. 3:**
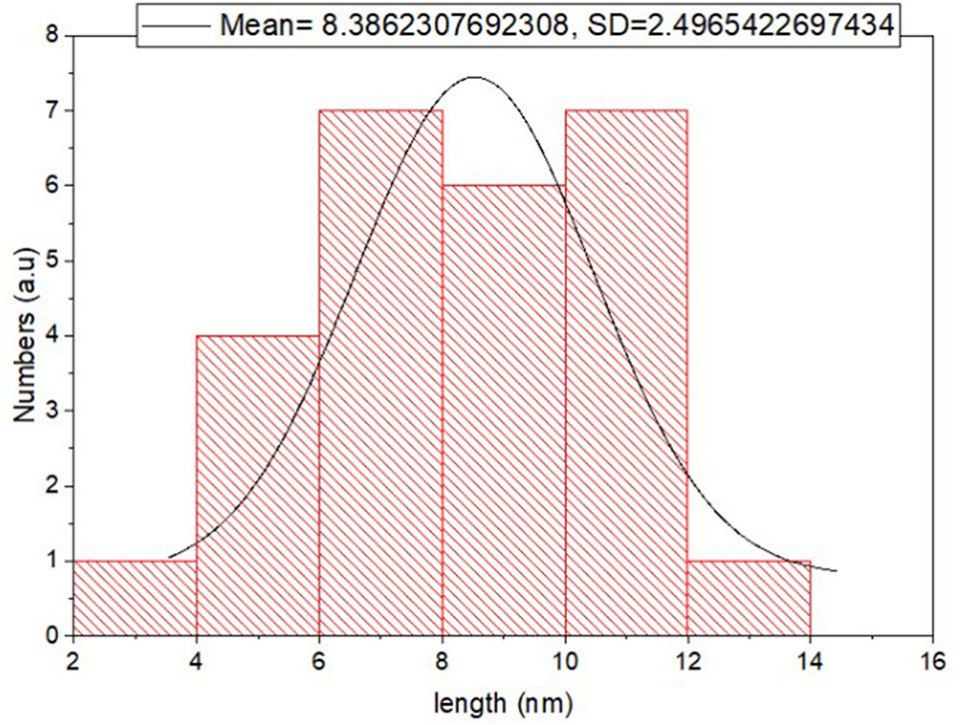
The size distribution histogram generated by TEM of biosynthesised Ag NPs using earthworm extract

The TEM micrographs visualize the characteristic spherical shape of Ag NPs as well as their size, ranging from 5 to 12 nm. The size range is consistent with the XRD result as well as the size reference in the literature (Figs. 2 and 3).

#### X-Ray diffraction

**Fig. 4:**
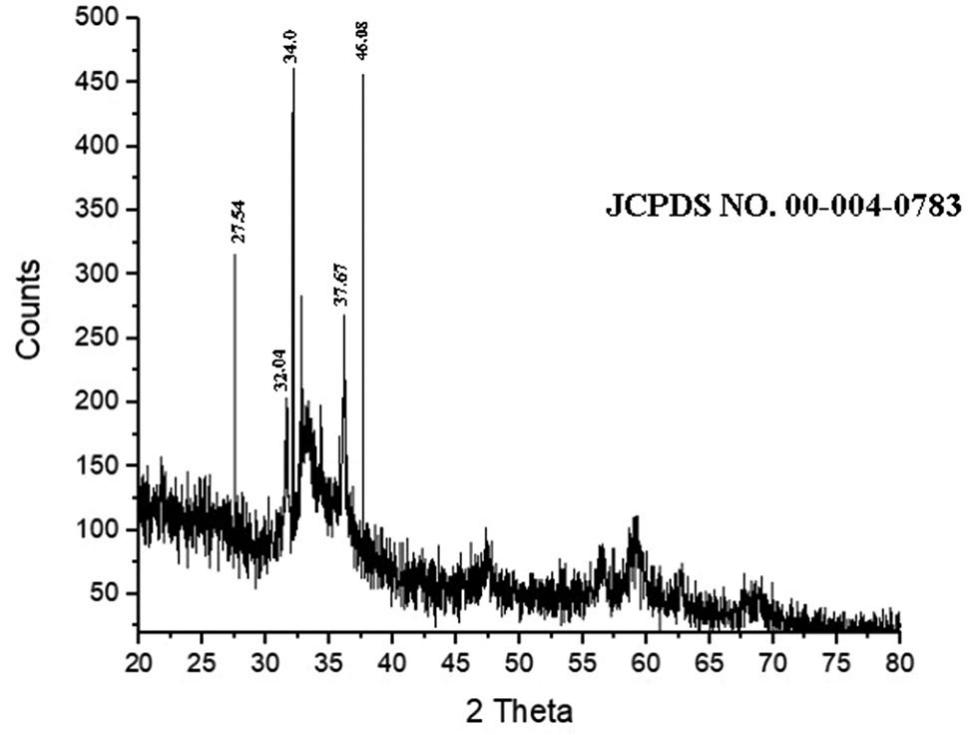
XRD pattern of biosynthesized Ag NPs using earthworm extract

The diffraction peaks obtained at 2θ were 27.54° ,32.04°, 34.0°,37.67° and 46.08°and indexed as (002), (111), (200), (220) and (311) respectively. These characteristic peaks indicate the presence of silver nanoparticles and confirm the face-centered cubic shape of Ag NPs (Fig. 4).

#### UV- visible spectrometry

**Fig. 5:**
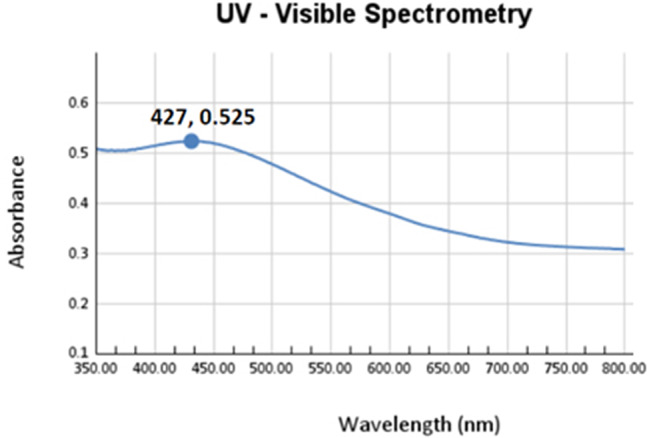
UV-Vis spectrometry of biosynthesised Ag NPs using earthworm extract

UV-Vis spectroscopy is a sensitive technique that can detect the formation of metallic nanomaterials. The ultraviolet spectra of Ag NPs indicated that Ag NPs have characteristic peaks between 400 and 430 (Fig. 5). The sample’s distinctive peak can be seen at 427 nm, supporting the presence of silver nanoparticles [26].

### Determination of MIC values of Ag NPs against tested pathogens

With a MIC value of 650 µg mL-1, Pseudomonas aeruginosa showed the lowest toxicity towards Ag NPs. Salmonella typhimurium is another Gram-negative bacteria having a MIC value of 1250 µg mL-1 that shows sensitivity to silver nanoparticles. A similar concentration of Ag Nps as calculated by the MIC (1250 µg mL-1) was given to gram-positive Staphylococcus aureus bacteria. On Gram-positive bacterium Listeria monocytogenes, Ag NPs showed no antibacterial action instead.

### Acute toxicity investigation of Ag nanoparticles in mice

The following formula was used to calculate LD50: LD50 = (M0 + M1)/2 = (10 + 100)/2 = 55 mg/kg. M0 was the highest dose of Ag NPs that did not cause mortality, and M1 was the lowest dose of Ag NPs that resulted in mortality. The Ag NPs dose is 1/10 of LD_50,_ which is 5.5 mg/kg.

### Serum biochemical parameters

As shown in Table 1, a significant (*p* < 0.05) increase in AST, ALT, and ALP is observed in septic mice compared to the control group. However, their levels significantly (*p* < 0.05) decreased in Ag NPs treated mice relevant to the septic group. While serum total protein and albumin in septic mice showed a significant (*p* < 0.05) decline compared to the control group. Ag NPs (5.5 mg/kg) treatment caused a significant (*p* < 0.05) increase in the content of total protein and albumin compared to the septic group. FIP injection showed a significant elevation (*P* < 0.05) in creatinine, urea, and uric acid levels of the septic group compared to control animals. Oral treatment of Ag NPs (5.5 mg/kg) caused a significant decrease in the levels of creatinine, urea, and uric acid to nearly normal levels compared to the septic group (Table 1).

**Table 1:** Antiseptic effect of Ag NPs on biochemical parameters of septic mice

Parameters	Groups		
		Sepsis	
	Control	Vehicle	Ag NPs
AST (U/mL)	82.1 ± 3.51 ^a^	135.8 ± 3.03 ^c^	122.11 ± 1.9 ^b^
ALT (U/mL)	36.28 ± 1.01 ^a^	70.41 ± 1.20 ^c^	45.93 ± 0.84 ^b^
ALP (U/L)	199.40 ± 6.74 ^a^	520.12 ± 25.33 ^c^	305.26 ± 15.92 ^b^
Urea (mg/dL)	29.69 ± 1.50 ^a^	47.57 ± 1.58 ^b^	32.60 ± 1.67 ^a^
Creatinine (mg/dL)	0.75 ± 0.02 ^a^	1.44 ± 0.04 ^c^	1.10 ± 0.05 ^b^
Uric Acid (mg/dL)	1.86 ± 0.07 ^a^	3.85 ± 0.20 ^b^	1.95 ± 0.14 ^a^
Total Protein (g/dL)	7.46 ± 0.12 ^b^	5.92 ± 0.04 ^a^	7.54 ± 0.18 ^b^
Albumin (g/dL)	4.97 ± 0.03 ^c^	4.03 ± 0.09 ^a^	4.65 ± 0.04 ^b^

Values are Mean ± SEM and values with different superscript letters are significantly different (*p* < 0.05)

### Oxidative stress markers in liver and kidney

As shown in Tables 2 and 3, the sepsis group generated a significant (*p* < 0.05) rise in the liver and kidney MDA and NO compared with the control group. The case was again reversed in the Ag NPs-treated mice as their hepatic and renal levels of MDA and NO declined as compared to the septic group. Relative to the control group, a significant (*p* < 0.05) decline was recorded in the hepatic and renal GSH, SOD, GST, and CAT of the septic group. Meanwhile, the Ag NPs-treated mice showed a significant (*p* < 0.05) increase in all four parameters.

**Table 2:** Antiseptic effect of Ag NPs on liver oxidative stress biomarkers of septic mice

Parameters	Groups		
		Sepsis	
	Control	Vehicle	Ag NPs
GSH (mM/g.tissue)	1.02 ± 0.03 ^c^	0.50 ± 0.02 ^a^	0.69 ± 0.03 ^b^
SOD (U/g.tissue)	92.91 ± 3.70 ^a^	53.38 ± 2.74 ^b^	70.65 ± 1.93 ^c^
GST (µM/g.tissue/min)	9.23 ± 0.34 ^a^	6.80 ± 0.32 ^b^	8.51 ± 0.23 ^b^
MDA (nM/g.tissue)	1.35 ± 0.03 ^a^	3.18 ± 0.31^c^	2.056 ± 0.13 ^b^
NO (µM/g.tissue)	298.68 ± 10.92 ^a^	801.37 ± 13.84 ^c^	712.80 ± 20.39 ^b^
CAT (U/min/g.tissue)	448.94 ± 14.28 ^c^	246.95 ± 11.57 ^a^	377.81 ± 20.80 ^b^

Values are Mean ± SEM and values with different superscript letters are significantly different (*p* < 0.05)

**Table 3:** Antiseptic effect of Ag NPs on kidney oxidative stress biomarkers of septic mice

Parameters	Groups		
		Sepsis	
	Control	Vehicle	Ag NPs
GSH (mM/g.tissue)	1.76 ± 0.08 ^a^	0.72 ± 0.05 ^b^	1.23 ± 0.07 ^c^
SOD (U/g.tissue)	195.81 ± 5.17 ^c^	101.74 ± 5.17 ^a^	164.60 ± 6.48 ^b^
GST (µM/g.tissue/min)	5.08 ± 0.10 ^c^	3.11 ± 0.02 ^a^	3.96 ± 0.08 ^b^
MDA (nM/g.tissue)	1.12 ± 0.04 ^a^	3.24 ± 0.07 ^c^	1.53 ± 0.09 ^b^
NO (µM/g.tissue)	267.20 ± 8.08 ^a^	418 ± 7.87 ^c^	336.40 ± 12.78 ^b^
Catalase (U/min/g.tissue)	506.81 ± 21.5 ^c^	226.88 ± 12.82 ^a^	369.33 ± 6.69 ^b^

Values are Mean ± SEM and values with different superscript letters are significantly different (*p* < 0.05)

### Histopathological examination

#### Kidney histopathology

**Fig. 6:**
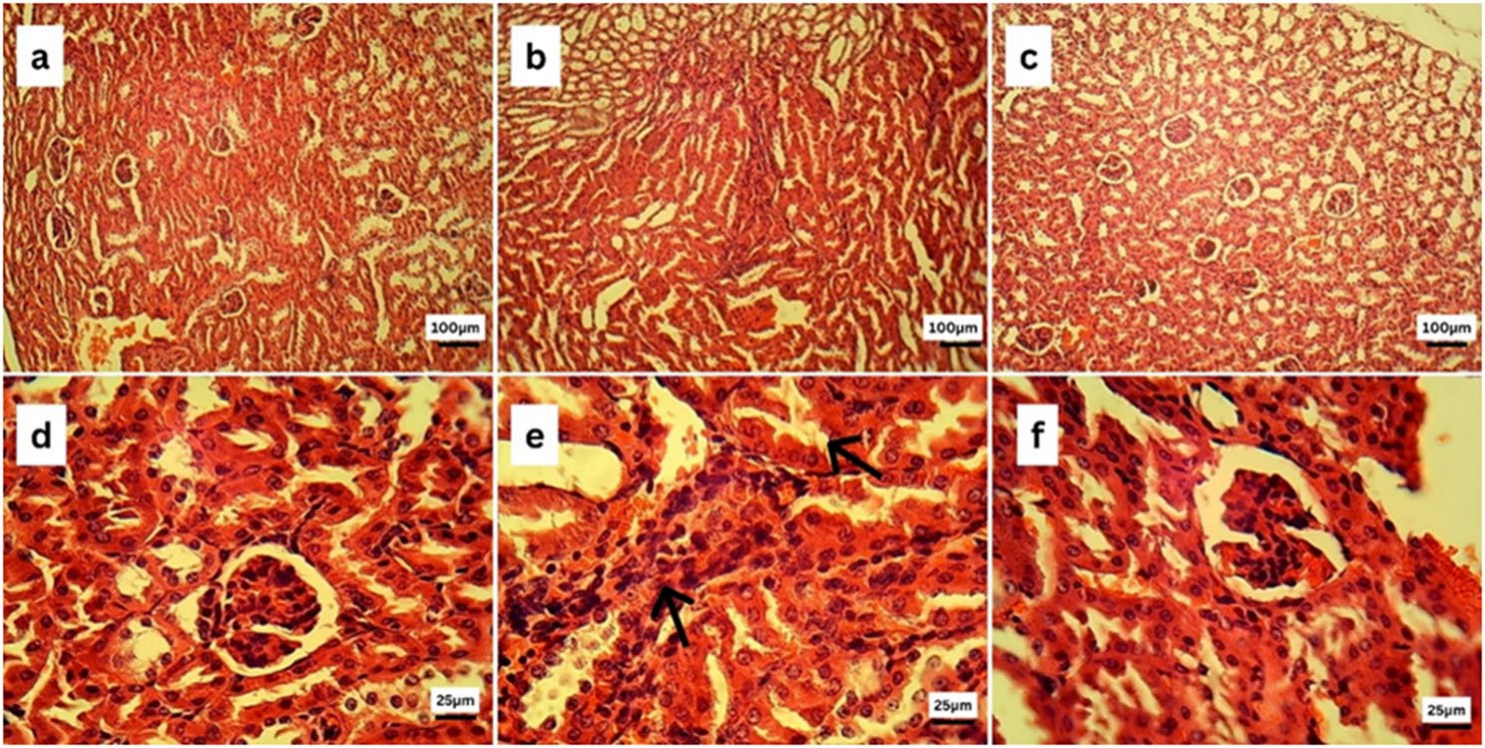
Photomicrographs of kidneys of the control (**a**, **d**), septic (**b**, **e**), and Ag NPs (**c**, **f**) groups stained by hematoxylin and eosin(H&E). Kidney sections of septic mice showing focal leukocytic cell infiltrations (arrow) and necrosed renal tubules (arrows)

Figure 6 illustrates a histopathological examination of kidney tissue from the control group (6a) and (6d) revealed the absence of any histopathological alterations in both the renal cortex and medulla. Glomerulus with abundant capsular space and tubules were histologically normal. On the contrary, the kidneys of septic mice (6b) and (6e) showed interstitial mononuclear inflammatory cell infiltrations and necrosed renal tubules. Interestingly, the kidney sections of the Ag NPs treated group (6c) and (6f) showed apparently normal renal cortex.

#### Liver histopathology

**Fig. 7:**
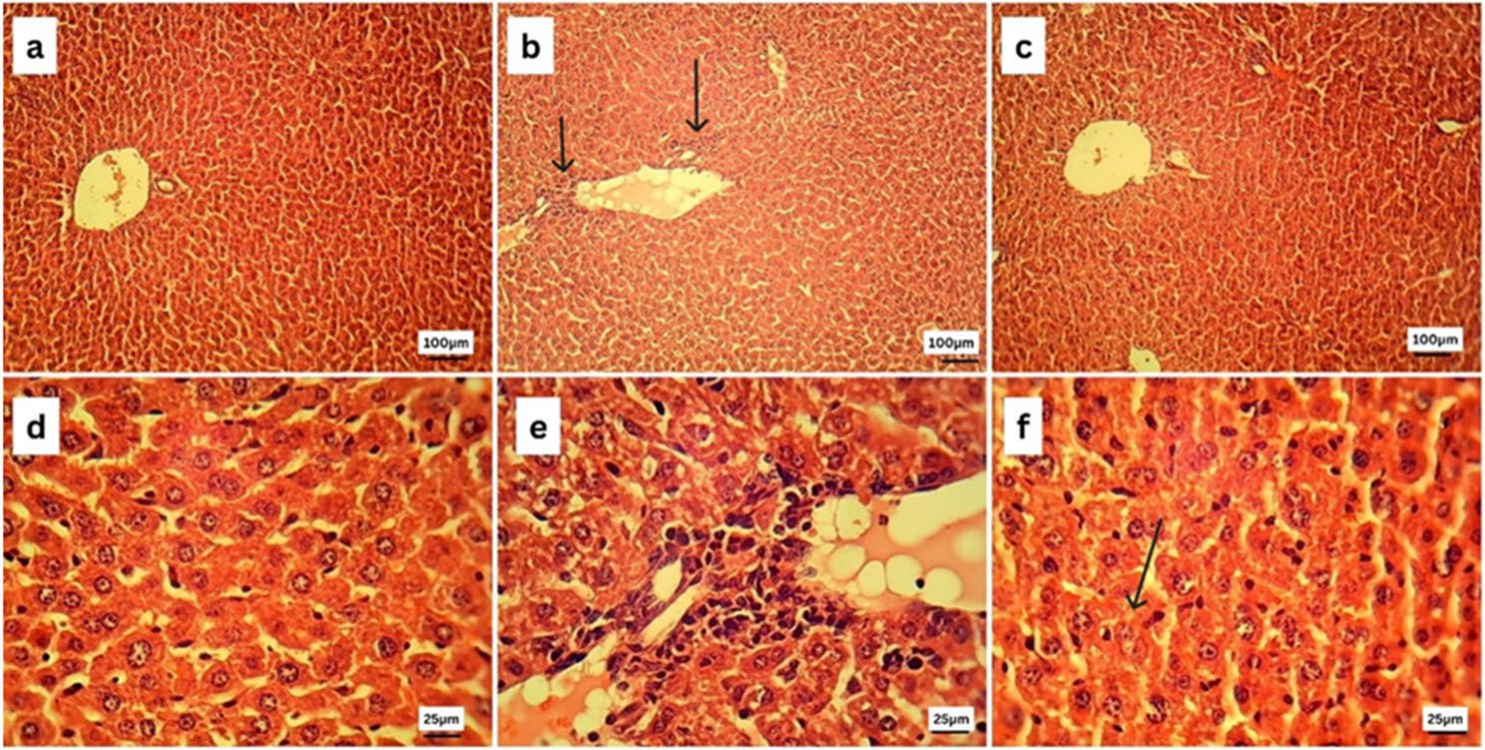
Photomicrographs of liver sections of the control (**a**, **d**), septic (**b**, **e**), and the Ag NPs (**c**, **f**) groups stained by hematoxylin and eosin (H&E). Liver sections of the septic group showed portal infiltration with mononuclear inflammatory cells (arrows). Liver sections of the Ag NPs group showed improvement in the central vein structure (arrow)

Figure 7 illustrates a histopathological examination of liver tissue from the control group (7a) and (7d), revealing the normal histological structure of hepatic parenchyma; both portal and centrilobular hepatocytes were normal that have rounded vesicular nuclei and appeared polygonal in shape. Sinusoids and Kupffer’s cells appear to be normal as well. On the contrary, the liver of septic mice (7b) and (7e) showed portal infiltration with mononuclear inflammatory cells. Interestingly, liver sections of the Ag NPs treated group (7c) and (7f) showed apparently normal central vein structure and normal hepatocyte, sinusoid, and Kupffer’s cell structure, with fewer necrotic cells being present.

## Discussion

Sepsis is an epidemic that impacts numerous parenchymatous organs and progresses gradually [27]. It is brought on by a serious bacterial infection, in particular one that arises in the abdominal cavity [28]. During sepsis, the liver is in charge of controlling the intense systemic inflammation that occurs by eliminating pathogenic mediators from the bloodstream [29]. The ongoing study hypothesised that hepatic damage could be caused by an increase in proinflammatory cytokines in response to polymicrobial infection [30]. Furthermore, the current study found that the septic induction caused significant hepatocellular damage in untreated septic mice, as evidenced by a substantial increase in activities of their liver enzymes (ALT, AST, and ALP) and a reduction in total protein, serum albumin as well. Serum albumin is a simple indicator of nutritional status and plays an important role in physiological homeostasis [31]. Our results showed hypoalbuminemia in septic mice compared to the control one. Sepsis patients frequently experience capillary leakage, reperfusion injury, tissue ischemia, and inflammatory responses, all of which can lead to hypoalbuminemia, which is a distinct indicator of prognosis in patients with sepsis and septic shock [28]. Notably, the administration of Ag NPs retained albumin, total protein, and activity of liver enzymes to the normal level. The aforementioned results were corroborated by histopathologic analysis, which revealed that Ag NPs treatment maintained the septic mice liver. The hepatoprotective effect of Ag NPs may be attributed to their antioxidant properties [32].

The kidney is a frequently impaired organ across sepsis, which increases the risk of death [33]. The renal functions were evaluated in the current study by measuring serum creatinine, urea, and uric acid concentrations. The creatinine level, which can partially determine the glomerular filtration rate as a consequence of solute eradication by the kidney, can be utilised for diagnosing acute kidney injury [34]. Elevation of blood urea is also a significant marker of renal failure [35]. The role of uric acid in the septic process is still debatable; however, as a molecule, it may act as a “marker for the cellular crisis” that indicates disorders in the processes responsible for cellular protein catabolism and energetic metabolism [36]. This would serve as a gauge for the state and progression of sepsis. As shown in our study, the sepsis induction of mice exhibited a significant increase in creatinine, urea, and uric acid contents as related to the control group. Several studies stated alterations in hemodynamics of the renal cortex or renal parenchymal injury could be the reason for an increase in plasma creatinine activity [37]^,^ [38]^,^ [39]. According to studies, changes in the microcirculation caused hypoxia and ischemia while also triggering endothelial xanthine oxidase. These procedures increased the production of uric acid [40]^,^ [41]. Intriguingly, the administration of the Ag NPs retrieved the creatinine, urea, and uric acid content and retained kidney function, as shown by the histopathology analysis of the kidneys in the treated septic mice. This may be attributed to the anti-inflammatory properties of Ag NPs. The total phenolic and flavonoid compounds strongly support the high anti-inflammatory activity of Ag NPs of earthworm whole extract [14, 42].

Oxidative stress is one of the most critical mechanisms related to a wide variety of disorders, including infection and sepsis. When the equilibrium between the body’s internal antioxidant system and both reactive oxygen species (ROS) and reactive nitrogen species (RNS) is disrupted, this can lead to oxidative stress in the body [43]. Sepsis is characterised by a state of hyper-oxidative stress that attacks endothelial cells directly and alters oxygen consumption, thereby fostering organ failure [44].

Malondialdehyde (MDA) is one of the extremely reactive metabolic byproducts of lipid peroxidation, in which free oxygen radicals influence tissues. Nitric oxide (NO) is an essential mediator in biochemical reactions and is produced by numerous cell types in response to cytokine stimulation [45]. In line with prior findings, fecal slurry injection resulted in a considerable elevation in MDA and NO levels. Sepsis leads to enhanced ROS and RNS burden by uncoupled oxidative phosphorylation and depleted antioxidant stores. The net result is accumulated damage to lipids, proteins, and nucleic acids that may inhibit cellular function. Oxidative injury to lipids within plasma and mitochondrial membranes can alter permeability and impair membrane-bound receptors and enzymes. MDA and other reactive aldehydes are among the toxic species that can disrupt protein structure and function [45].

Concerning the endogenous antioxidant components, the current investigation demonstrated that fecal slurry injection resulted in the depletion of the enzymatic and nonenzymatic antioxidants. These endogenous antioxidants included GSH, CAT, SOD, and GST in both the liver and the kidneys. The decreased GSH level in the septic animals might be due to increased scavenging of ROS that was produced because of the inflammatory and necrotic states [46]. GSH depletion may cause H_2_O_2_ to accumulate in tissues and then diffuse into the bloodstream, causing microvascular malfunction and organ failure in septic shock [47]. Reduced levels of GSH in the liver and kidney may cause the suppression of GST’s activities, as GSH is a substrate and cosubstrate in crucial enzymatic reactions [48]. Additionally, the production of sepsis stimulates an inflammatory response, encouraging the release of pro-inflammatory cytokines (IL-6 and TNF-), which in turn increases the amount of reactive oxygen species [10].

Curiously, we revealed that the treatment with the biogenic Ag NPs showed a powerful antioxidant effect via the significant decrease in MDA and NO levels and the increase in antioxidant content (GSH, CAT, SOD, and GST). Ag NPs possess a broad spectrum of antibacterial, antifungal, and antiviral properties. They can penetrate bacterial cell walls, changing the structure of cell membranes and even resulting in cell death [49]. In the same line, Ag NPs act as an anti-inflammatory agent by elevating the apoptosis of inflammatory cells and declining the level of pro-inflammatory cytokines [50]. Moreover, Ag NPs have powerful antioxidant abilities, scavenging free radicals and enhancing the endogenous antioxidant defense system.

## Conclusion

**Fig. 8:**
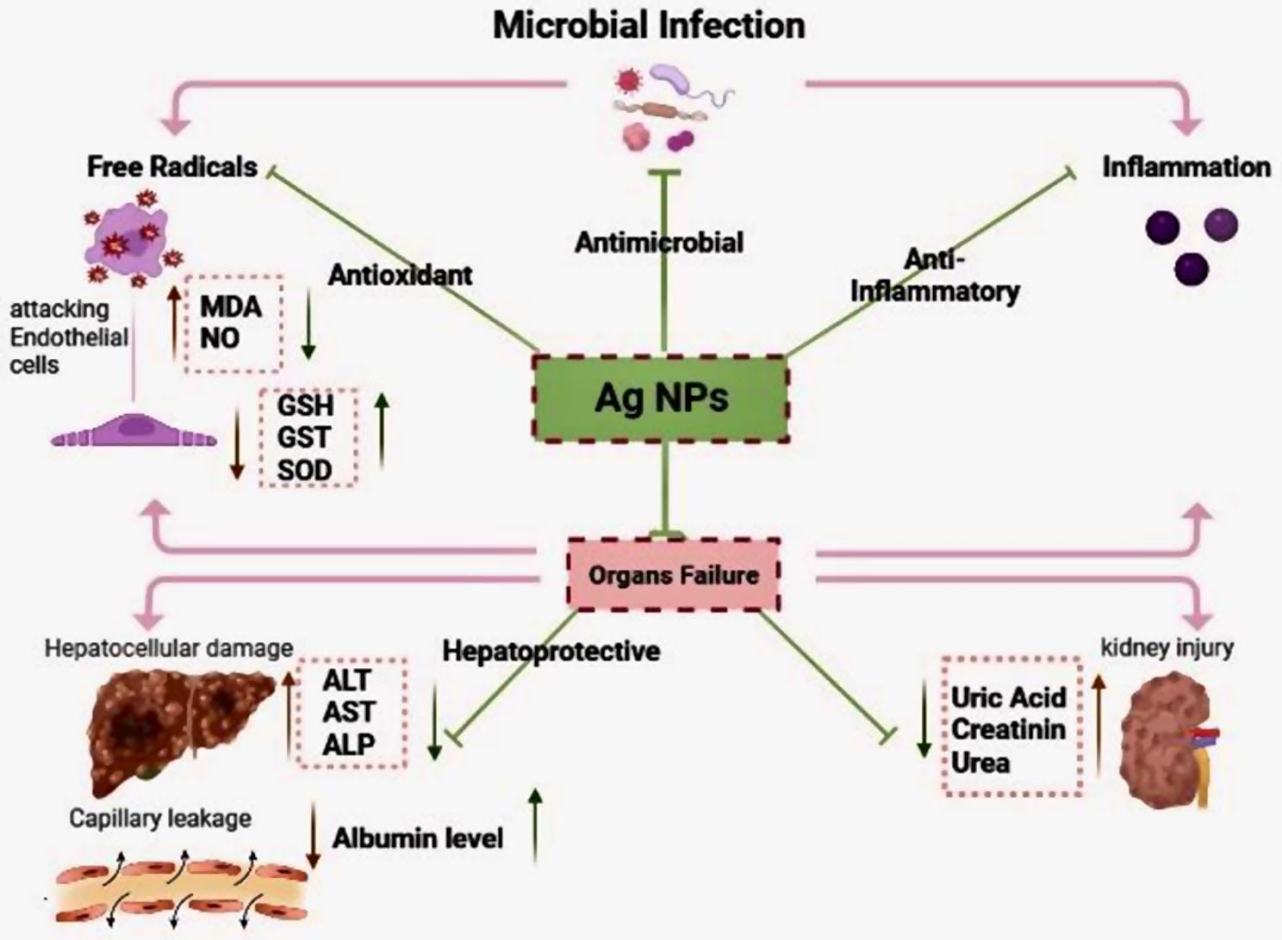
Schematic diagram showing the efficacy of silver nanoparticles synthesised from earthworm extract on serum and tissue compartments via multiple pathways against sepsis

This study demonstrated the mechanism by which Ag NPs can effectively treat sepsis in mice (Fig. 8). AgNPs act against microbial infection through three main actions: antioxidant, antimicrobial, and anti-inflammatory effects. Ag NPs’ antioxidant action includes detoxifying free radicals that attack endothelial cells, decreasing MDA and NO levels, and increasing the antioxidant system, including GST, GSH, and SOD. The antimicrobial action of Ag NPs includes generating Ag ions, which bind to the bacteria’s cell body and destroy it. AgNPs act as an anti-inflammatory agent by increasing inflammatory cell apoptosis and decreasing pro-inflammatory cytokine levels. In septic mice, the biogenic Ag NPs improved kidney and liver parameters by enhancing liver enzymes and decreasing kidney metabolites. Histopathological examination results confirm the efficacy of biogenic AgNPs in improving hepatic and renal architecture. These results indicate that biogenic Ag NPs could potentially serve as a novel and effective strategy for preventing sepsis.

## Data Availability

No datasets were generated or analysed during the current study.
